# Gut microbiota modulate intestinal inflammation by endoplasmic reticulum stress-autophagy-cell death signaling axis

**DOI:** 10.1186/s40104-025-01196-8

**Published:** 2025-05-02

**Authors:** Feiyang He, Yi Zheng, Mabrouk Elsabagh, Kewei Fan, Xia Zha, Bei Zhang, Mengzhi Wang, Hao Zhang

**Affiliations:** 1 Laboratory of Metabolic Manipulation of Herbivorous Animal Nutrition, College of Animal Science and Technology, Yangzhou University, Yangzhou, 225009 P. R. China; 2 Joint International Research Laboratory of Agriculture and Agri-Product Safety, the Ministry of Education of China, Yangzhou University, Yangzhou, 225009 P. R. China; 3 Key Laboratory of Fujian Universities Preventive Veterinary Medicine and Biotechnology, Longyan University, Longyan, 364012 P. R. China; 4 Department of Animal Production and Technology, Faculty of Agricultural Sciences and Technologies, Niğde Ömermer Halisdemir University, Nigde, 51240 Turkey; 5 State Key Laboratory of Sheep Genetic Improvement and Healthy Production, Xinjiang Academy of Agricultural Reclamation Science, Shihezi, 832000 P. R. China

**Keywords:** Autophagy, Cell death, Endoplasmic reticulum stress, Gut microbes, Intestinal inflammation

## Abstract

The intestinal tract, a complex organ responsible for nutrient absorption and digestion, relies heavily on a balanced gut microbiome to maintain its integrity. Disruptions to this delicate microbial ecosystem can lead to intestinal inflammation, a hallmark of inflammatory bowel disease (IBD). While the role of the gut microbiome in IBD is increasingly recognized, the underlying mechanisms, particularly those involving endoplasmic reticulum (ER) stress, autophagy, and cell death, remain incompletely understood. ER stress, a cellular response to various stressors, can trigger inflammation and cell death. Autophagy, a cellular degradation process, can either alleviate or exacerbate ER stress-induced inflammation, depending on the specific context. The gut microbiome can influence both ER stress and autophagy pathways, further complicating the interplay between these processes. This review delves into the intricate relationship between ER stress, autophagy, and the gut microbiome in the context of intestinal inflammation. By exploring the molecular mechanisms underlying these interactions, we aim to provide a comprehensive theoretical framework for developing novel therapeutic strategies for IBD. A deeper understanding of the ER stress-autophagy axis, the gut microbial-ER stress axis, and the gut microbial-autophagy axis may pave the way for targeted interventions to restore intestinal health and mitigate the impact of IBD.

**Graphical Abstract:**

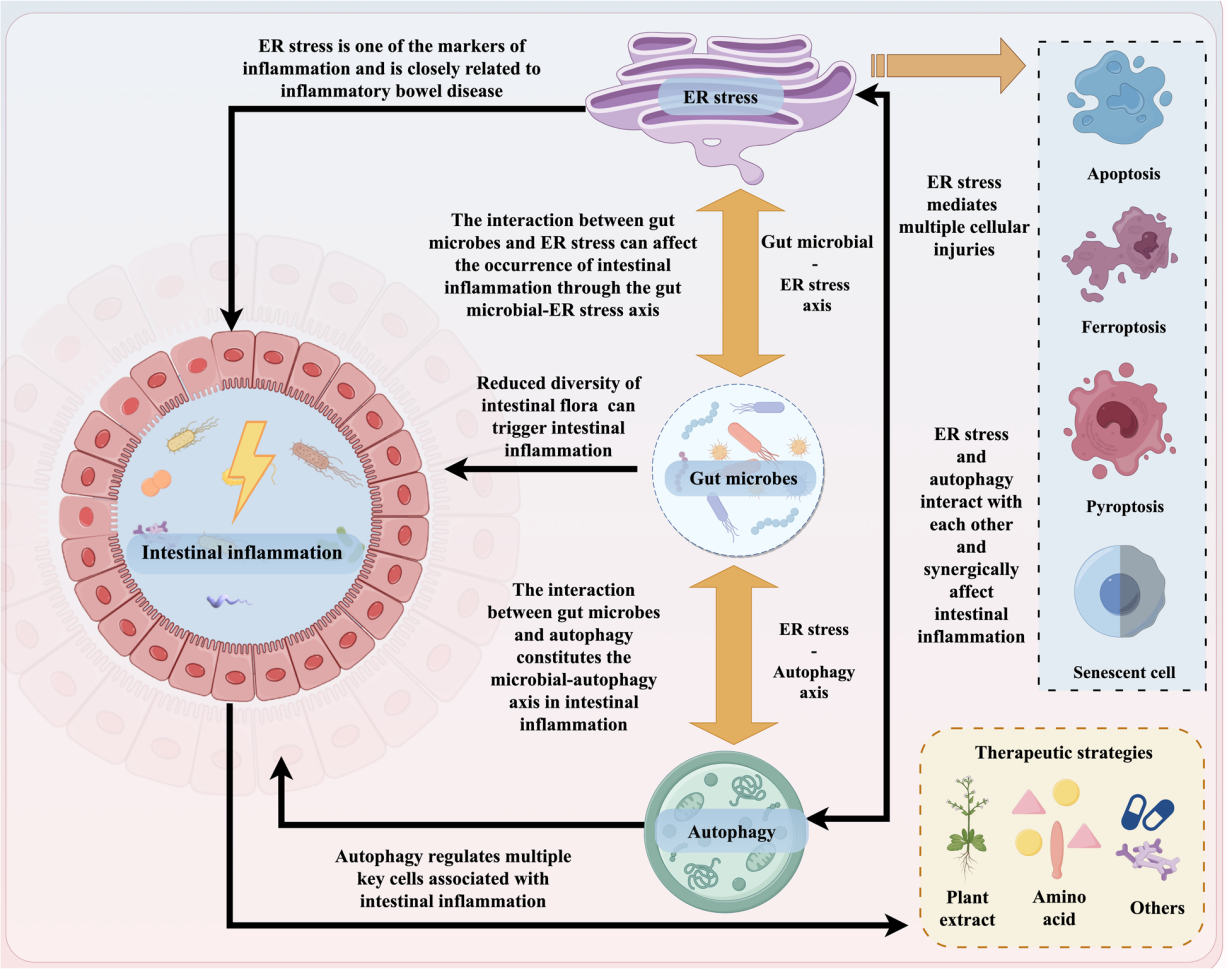

## Introduction

The intestine is a vital organ for digestion and absorption in animals. Its integrity is essential for maintaining the intestinal barrier [1]. Hypoxia and other stressors can increase intestinal permeability, leading to barrier dysfunction and systemic inflammation [2]. Inflammation is a physiological response to harmful stimuli, like pathogens or tissue damage. Inflammatory Bowel Disease (IBD), including Crohn’s disease (CD) and ulcerative colitis (UC), affects both animals and humans [3].

The endoplasmic reticulum (ER), the largest organelle in animal cells, is a complex network of membranes involved in protein processing, modification, and calcium homeostasis [4]. Persistent ER stress can damage other organelles, leading to cell death through various mechanisms, including autophagy, apoptosis, ferroptosis, pyroptosis, and senescence [5]. ER stress, often observed in intestinal epithelial cells, can trigger spontaneous intestinal inflammation. This stress has been identified in the ileal or colonic epithelium of IBD patients [6]. Autophagy is a cellular process that maintains intestinal health by degrading cellular components and regulating the gut microbiome [7]. Emerging research indicates that autophagy plays a crucial role in IBD [8]. By protecting cells from bacterial toxins, autophagy maintains cell survival. Impaired autophagy can lead to intestinal cell dysfunction, microbial imbalance, and uncontrolled inflammation [8]. The mammalian gut harbors trillions of bacteria, collectively known as the gut microbiota, which play a vital role in various aspects of health and disease [9]. Microbial imbalances, or dysbiosis, can promote the growth of harmful bacteria, worsening intestinal inflammation [10].

This review explores the role of ER stress in intestinal inflammation, focusing on its impact on cellular injury and its interaction with autophagy. We delve into the intricate relationship between gut microbiota, ER stress, and autophagy in modulating intestinal inflammation. Additionally, we discuss potential therapeutic strategies for mitigating gut inflammation and improving animal health.

## The connection between ER stress and the unfolded protein response

**Fig. 1:**
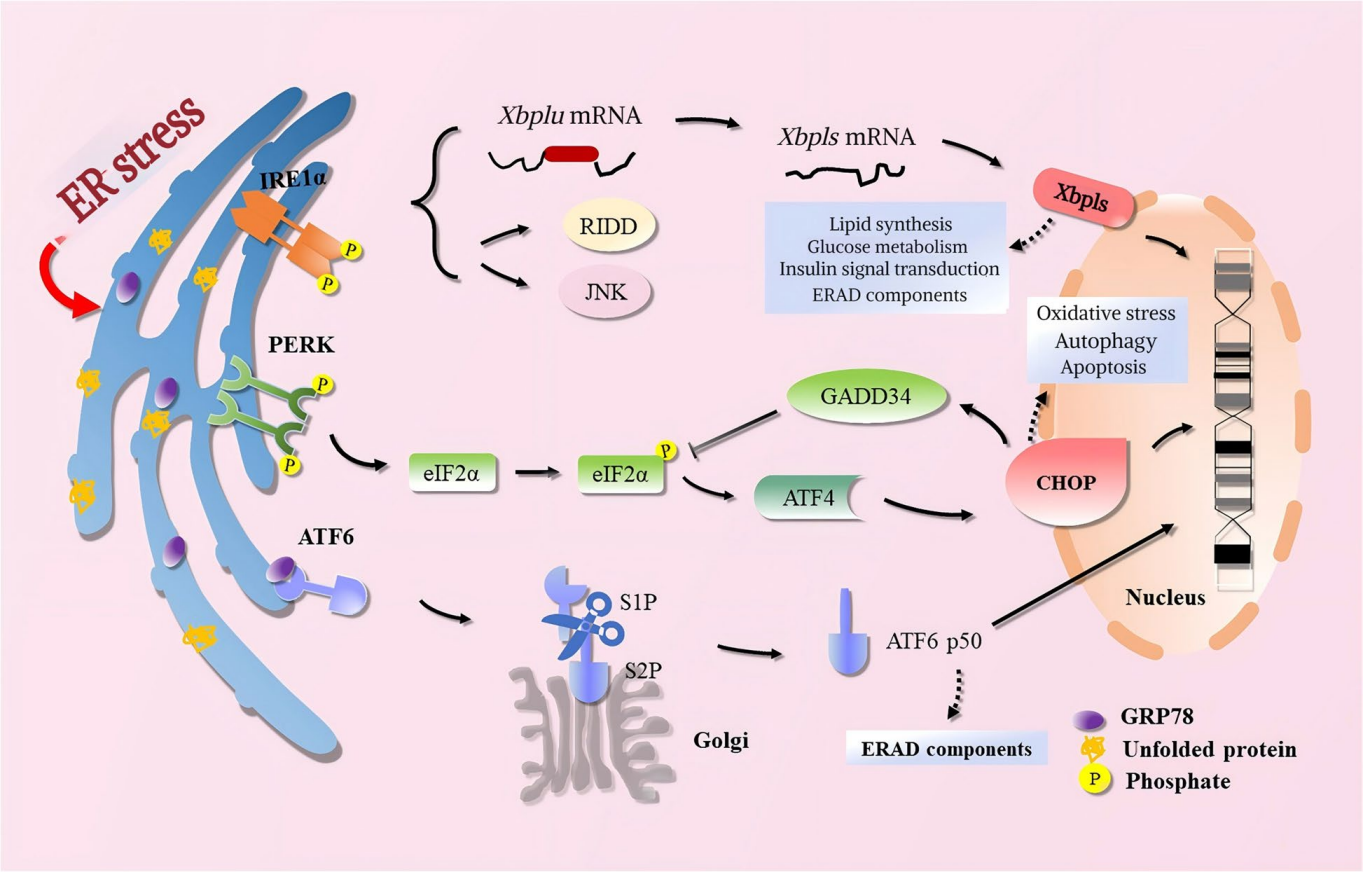
The UPR is a crucial cellular mechanism activated in response to ER stress, primarily triggered by the accumulation of misfolded or unfolded proteins. In mammals, this response involves three key sensing proteins: IRE1α, PERK, and ATF6. These proteins are initially kept inactive by binding to the chaperone GRP78/Bip. Upon ER stress, GRP78 dissociates from these sensors, allowing their activation and subsequent signaling. ATF, activating transcription factor; CHOP, C/EBP Homologous Protein; eIF2α, eukaryotic initiation factor 2 alpha; ER, endoplasmic reticulum; GRP78, glucose-regulated protein 78; IRE1α, inositol-requiring enzyme 1α; JNK, c-Jun N-terminal kinase; PERK, protein kinase R-like ER kinase; RIDD, regulated IRE1-dependent decay; S1P, site-1 protease; S2P, site-2 protease; UPR, unfolded protein response

Various physiological and pathological conditions, including temperature fluctuations, pH imbalances, DNA damage, oxidative stress, and hypoxia, can disrupt ER homeostasis, leading to significant cellular consequences. These disruptions often result in the accumulation of misfolded proteins, overwhelming the cell's protein folding capacity and triggering the unfolded protein response (UPR), causing ER damage and stress [11]. While this response is initially protective, chronic ER stress can result in cellular dysfunction and contribute to various diseases. The UPR can mitigate ER stress by activating ER-associated protein degradation. This involves upregulating molecular chaperones like Bip and glucose-regulated protein 78 (GRP78), which bind to misfolded proteins and facilitate their degradation, reducing the accumulation of harmful proteins [12]. Additionally, the UPR reduces the translation of mRNAs into proteins, preventing further protein buildup within the ER [13]. Persistent ER stress triggers the release of three key sensing proteins: Inositol-requiring enzyme 1α (IRE1α), protein kinase R-like ER kinase (PERK), and activating transcription factor (ATF) 6 (ATF6). These proteins are normally bound to GRP78/Bip but are released upon ER stress. The activated sensing proteins then initiate downstream signaling pathways to alleviate ER stress. These three proteins can act independently or cooperatively to mitigate the effects of ER stress [14] (Fig. 1).

The IRE1α signaling pathway, a highly conserved component of the UPR, is a transmembrane protein in the endoplasmic reticulum with two enzymatic activities: protein kinase and endoribonuclease [15–23]. PERK, a type I transmembrane protein located in the endoplasmic reticulum, is a key regulator of ER stress. Its serine/threonine kinase domain plays a crucial role in determining cell fate by activating downstream signaling pathways. During ER stress, PERK dissociates from Bip and dimerizes. This activated PERK phosphorylates eukaryotic initiation factor 2 alpha (eIF2α), reducing global protein translation and alleviating ER stress [24–27]. ATF6, a type II transmembrane protein, is another key sensor of ER stress. Unlike PERK and IRE1, ATF6 translocates to the Golgi apparatus upon ER stress. There, it undergoes a two-step proteolytic cleavage by site-1 protease (S1P) and S2P. This cleavage releases a 50-kDa cytosolic fragment of ATF6, known as ATF6f that translocates to the nucleus to activate the transcription of genes involved in the unfolded protein response [28–31].

In conclusion, the UPR is a cellular response to ER stress that aims to restore protein homeostasis. By activating three signaling pathways (IRE1α, PERK, and ATF6), the UPR can alleviate ER stress by reducing protein load and promoting protein folding. However, if the UPR is overwhelmed or prolonged, it can trigger cell death pathways, highlighting the delicate balance between cellular survival and demise under ER stress conditions.

## ER stress-mediated intestinal autophagy and cell death

ER stress plays a significant role in the pathophysiology of intestinal disorders, leading to various detrimental outcomes. Prolonged ER stress can have detrimental effects on intestinal cells, triggering various cell death pathways including autophagy, apoptosis, ferroptosis, pyroptosis, and senescence.

### ER stress-mediated autophagy

Autophagy, a conserved cellular degradation process, primarily involves macroautophagy, microautophagy, and chaperone-mediated autophagy (CMA). ER stress often triggers non-selective macroautophagy, which helps degrade misfolded proteins and alleviate cellular stress. While autophagy is essential for maintaining cellular homeostasis, excessive or dysregulated autophagy can contribute to cell death [32]. When the ER becomes overloaded with misfolded proteins, autophagy can be activated as a secondary response to degrade these proteins and alleviate ER stress [24]. The three major branches of the UPR, IRE1α, PERK, and ATF6, can regulate autophagy through various signaling pathways.

**Fig. 2:**
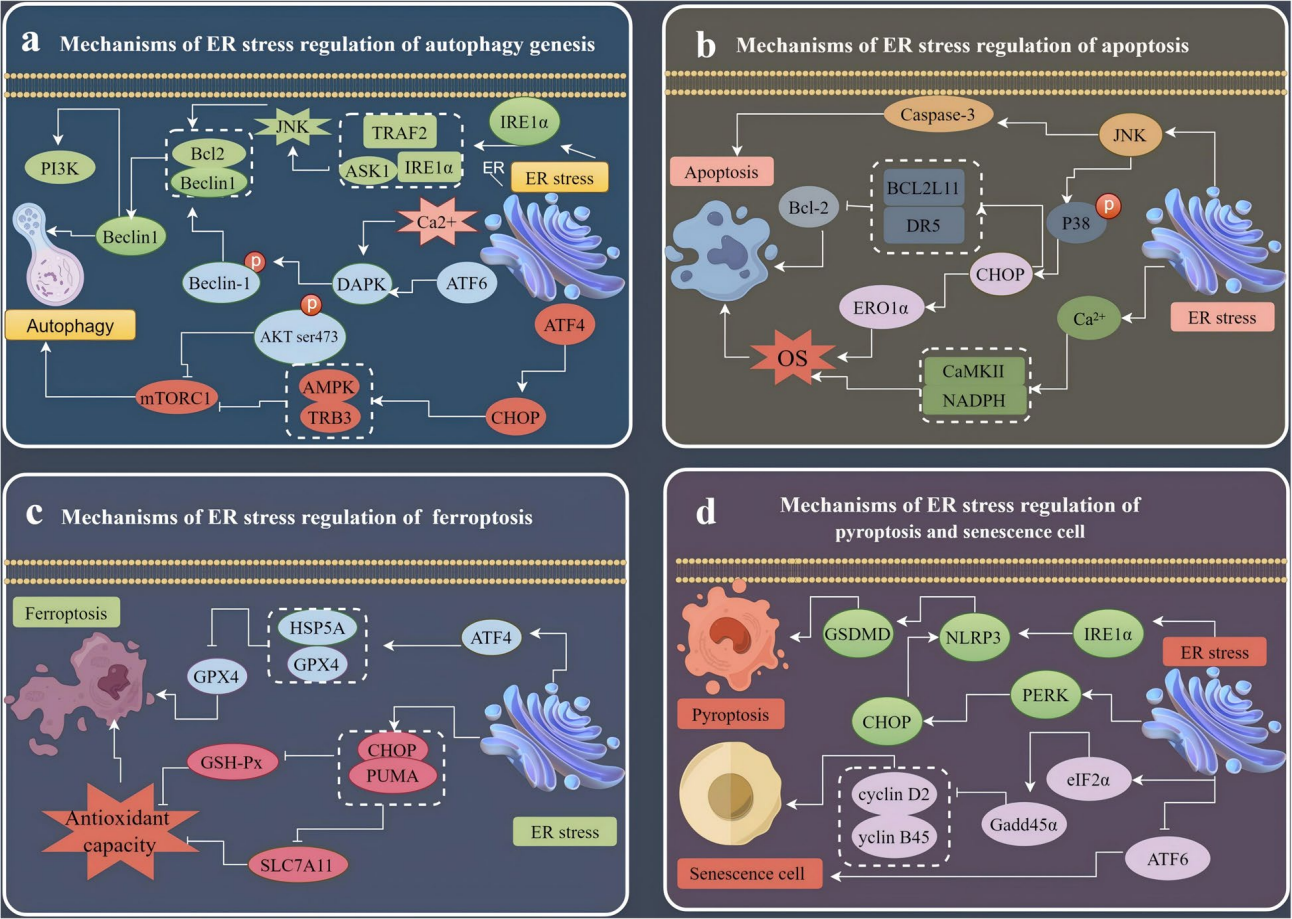
Under conditions of sustained ER stress, intestinal cells can activate various cell death pathways, including autophagy (**a**), apoptosis (**b**), ferroptosis (**c**), and pyroptosis and cellular senescence (**d**). Each of these pathways is regulated by distinct mechanisms influenced by ER stress. ASK, apoptosis signal regulating kinase-1; ATF, activating transcription factor; AMPK, adenosine monophosphate-activated protein kinase; Bcl2, B-cell lymphoma-2; CAMKII, calmodulin-dependent protein kinase II; CHOP, C/EBP Homologous Protein; DR5, death receptor 5; eIF2α, eukaryotic initiation factor 2 alpha; ER, endoplasmic reticulum; ERO1α, ER oxidoreductin 1 alpha; GPX4, glutathione peroxidase 4; HSP5A, heat shock protein family A; IRE1α, inositol-requiring enzyme 1α; JNK, c-Jun N-terminal kinase; mTORC1, mammalian target protein 1 of rapamycin; NLRP3, nucleotide-binding oligomerization domain-like receptor protein 3; PERK, protein kinase R-like ER kinase; PI3K, phosphatidylinositol 3-kinase; PUMA, p53 up-regulated modulator of apoptosis protein; TRB3, tribbles pseudokinase 3

The accumulation of unfolded proteins in the ER activates IRE1α through oligomerization and autophosphorylation, leading to the formation of signaling complexes with tumor necrosis factor receptor-associated factor 2 (TRAF2) and apoptosis signal-regulated kinase 1. This activation triggers Jun N-terminal kinase (JNK) signaling, which promotes autophagy by facilitating the release of Beclin1 from Bcl-2 and activating the phosphatidylinositol 3-kinase (PI3K) complex [33]. Additionally, activating ATF4 upregulates autophagy-related genes like *Atg12* and *Atg5* [34]. Concurrently, ATF4 can promote the expression of pro-apoptotic factors like C/EBP homologous protein (CHOP) which further promotes autophagy by inhibiting mammalian target protein 1 of rapamycin (mTORC1) through activating downstream pathways involving adenosine monophosphate-activated protein kinase (AMPK) and Tribbles homologous protein 3 [35]. Upon prolonged ER stress, ATF6 becomes activated and can induce autophagy by activating death-associated protein kinase (DAPK), which phosphorylates Beclin1, facilitating its release from the Bcl-2 complex [36]. Additionally, ATF6 can inhibit AKT phosphorylation at Ser473 by upregulating GRP78, leading to reduced mTOR activity and further activation of autophagy [37]. Furthermore, the release of calcium ions (Ca^2+^) from the ER via inositol trisphosphate receptors activates DAPK, promoting Beclin1 phosphorylation and inducing autophagy [38]. Through these mechanisms, cells can effectively manage ER stress by degrading misfolded proteins, thus maintaining cellular homeostasis. However, if ER stress persists or becomes excessive, it may lead to cell death pathways alongside autophagy. Understanding these processes is crucial for developing therapeutic strategies targeting ER stress-related diseases (Fig. 2a).

### ER stress-mediated apoptosis

Apoptosis, also known as programmed cell death, is a highly organized process that plays a crucial role in various biological contexts, including organ development, tissue homeostasis, and the immune response [39]. ER stress can trigger apoptotic cell death through several interconnected pathways. Activated JNK and p38 mitogen-activated protein kinase (p38 MAPK) pathways, induced by ER stress, promote the expression of pro-apoptotic genes such as *Caspase-3*, *BCL2L11*, and death receptor 5, while downregulating anti-apoptotic *Bcl-2* [40–42]. These pathways promote the expression of pro-apoptotic genes while inhibiting survival signals, ultimately leading to programmed cell death. Additionally, *CHOP*, a transcription factor activated by ER stress, can induce oxidative stress by upregulating ER oxidoreductin 1 alpha (ERO1α) and further promote apoptosis [43, 44]. Increased intracellular Ca^2+^ levels during ER stress can also contribute to apoptosis by activating calcium/calmodulin-dependent protein kinase II and NADPH oxidase [45]. Studies in intrauterine growth-restricted lambs have shown that ER stress in colonic cells induces apoptosis through both mitochondrial and death receptor pathways. This is characterized by increased expression of ER stress markers like GRP78 and CHOP, activation of caspases, and altered expression of apoptosis regulators such as Bax and Bcl-2 [46] (Fig. 2b).

### ER stress-mediated ferroptosis

Ferroptosis is a form of regulated cell death characterized by iron-dependent lipid peroxidation. Unlike apoptosis and autophagy, it does not involve typical morphological changes but is associated with mitochondrial alterations, such as increased membrane density and contraction [47, 48]. ATF4, an ER stress-activated transcription factor, can induce ferroptosis by upregulating heat shock protein family A (HSP5A), also known as GRP78. HSP5A stabilizes glutathione peroxidase 4 (GPX4), a crucial antioxidant enzyme that protects cells from lipid peroxidation. When GPX4 activity is compromised, cells become more susceptible to ferroptosis [49]. ER stress, particularly through the PERK-eIF2α-ATF4 pathway which leads to increased levels of CHOP, can contribute to ferroptosis. Upregulated CHOP, a downstream target of ATF4, can bind to p53 up-regulated modulator of apoptosis and downregulate SLC7A11 and GPX4, leading to decreased antioxidant capacity and increased susceptibility to ferroptosis [50, 51] (Fig. 2c).

### ER stress-mediated pyroptosis

Pyroptosis, a form of programmed cell death, is triggered by inflammatory vesicles and activated caspases [52]. Caspase-1 activation, induced by pathogens or other stimuli, cleaves gasdermin D (GSDMD), disrupting the cell membrane and leading to cell swelling and rupture [53]. Simultaneously, pro-inflammatory cytokines interleukin (IL)-1beta (IL-1β) and IL-18 mature and are released, initiating an inflammatory response [54]. In the ER stress pathway, IRE1α activates the nucleotide-binding oligomerization domain-like receptor protein 3 (NLRP3) pathway, promoting GSDMD release and exacerbating pyroptosis [55]. LPS-induced ER stress activates IRE1α and PERK, upregulating CHOP expression, activating NLRP3 inflammatory vesicles, and inducing cellular pyroptosis [56]. Excessive Cu^2+^ intake has been shown to increase GRP78 and caspase-1 expression, upregulating ER stress and pyroptosis-related gene and protein expression in porcine jejunum epithelium [57] (Fig. 2d).

### ER stress-mediated cellular senescence

Cellular senescence is an irreversible cell cycle arrest accompanied by increased secretion of inflammatory factors, induced by various stress responses, including ER stress, mitochondrial dysfunction, and oxidative damage [58]. Imbalances in ER protein homeostasis can induce cellular senescence, particularly through ER reductive stress mechanisms. In an ER-specific reductive stress cell model, increased senescence levels were correlated with elevated nitrosylation of Ero1α at cysteine residues Cys166 and Cys131. This nitrosylation reduced Ero1α’s oxidative enzyme activity, an essential enzyme for oxidative protein folding in the ER, leading to ER reductive stress and subsequent cellular senescence [59]. The growth arrest and DNA damage-inducible protein 45 alpha (GADD45α) inhibits cyclin D2 and cyclin B1 expression via the eIF2α pathway, leading to cell cycle arrest at the G1/S and G2/M phases. While GADD45α plays a protective role by inducing cell cycle arrest during stressful conditions, its presence can also promote apoptosis when cellular damage is irreparable. Interestingly, reducing GADD45α expression attenuates ER stress-induced cell death, suggesting that inhibiting GADD45α may enhance cell survival under conditions that would typically lead to cell death [60]. Additionally, ATF6 knockout mesenchymal stem cells exhibit multiple organelle dysfunction and accelerated cellular senescence [61]. Furthermore, impaired or imbalanced autophagy promotes pathological senescence [62]. Wang et al. [63] found that activation of ATF4 and CHOP induces cellular senescence through autophagy (Fig. 2d).

## ER stress mediates intestinal inflammation

**Fig. 3:**
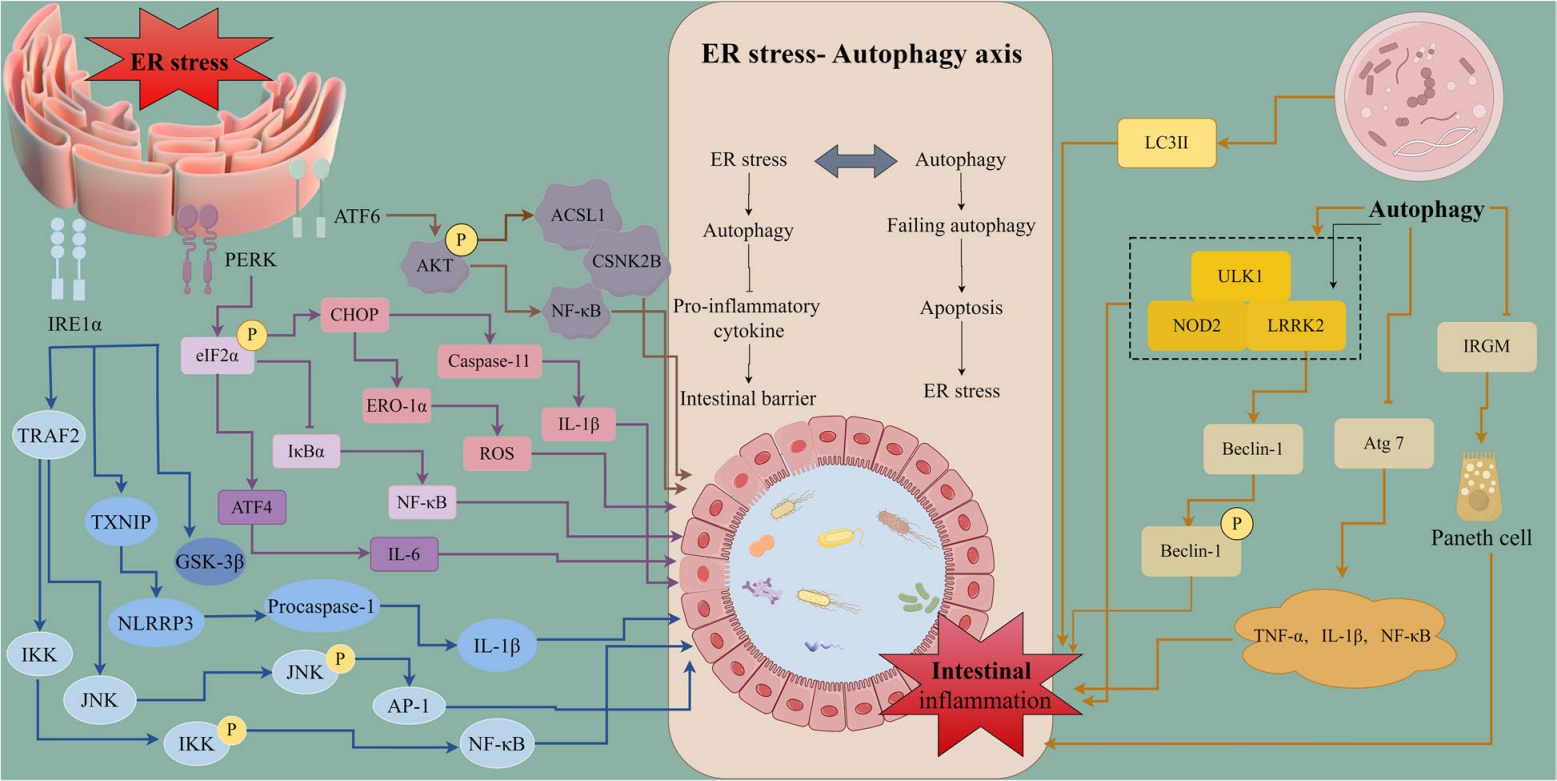
ER stress is a well-established contributor to the pathogenesis of IBD. The three key branches of the UPR—PERK, IRE1α, and ATF6—act as signaling pathways mediating the inflammatory response in IBD. Autophagy, a cellular self-degradation process, plays a critical role in the development of IBD and regulates various factors associated with intestinal inflammation. Interestingly, ER stress can induce autophagy through multiple pathways, including the UPR and Akt signaling. This intricate interplay between ER stress and autophagy forms the ER stress-autophagy axis, which exerts a synergistic effect on intestinal inflammation and the development of intestinal barrier function. ATF, activating transcription factor; AP-1, activator protein-1; CHOP, C/EBP homologous protein; CSNK2B, casein kinase 2 beta; eIF2α, eukaryotic initiation factor 2 alpha; ER, Endoplasmic reticulum; ERO1α, ER oxidoreductin 1 alpha; IBD, inflammatory bowel disease; GSK-3β, glycogen synthase kinase 3 beta; IKK, inhibitor of kappa B kinase; IL, interleukin; IRE1α, inositol-requiring enzyme 1α; IRGM, immunity-related GTPase M; JNK, c-Jun N-terminal kinase; LRRK2, leucine-rich repeat kinase 2; NF-κB, nuclear factor kappa-light-chain-enhancer of activated B cells; NOD2, nucleotide-binding oligomerization domain 2; TRAF2, tumor necrosis factor receptor-associated factor 2; TXNIP, thioredoxin interacting protein; UPR, unfolded protein response

Intestinal inflammation is a comprehensive concept that encompasses the inflammatory response of the intestinal mucosa and its surrounding tissues due to a variety of etiologies. IBD is part of intestinal inflammation. Excessive inflammation can impair gut morphology and function, leading to ER stress, which is a hallmark of inflammation associated with various diseases, including IBD [64, 65]. You et al. [66] integrated transcriptional programs with genome-scale Clustered Regularly Interspaced Short Palindromic Repeats screening to functionally dissect the UPR pathway and identify QRICH1 as a key effector of the PERK-eIF2α axis. QRICH1 controls a transcriptional program associated with translation and secretory networks that are specifically upregulated in inflammatory pathologies, which dictates cell fate in response to pathological ER stress [66]. The UPR’s three branches (PERK, IRE1α, and ATF6) mediate the inflammatory response in crosstalk with each other and with nuclear factor kappa-light-chain-enhancer of activated B cells (NF-κB) and activator protein-1 (AP-1), influencing the expression of pro-inflammatory cytokines like IL-8, IL-6, and TNF-α [67–72]. Understanding how ER stress triggers intestinal inflammation and disrupts the intestinal barrier is crucial for elucidating the mechanisms underlying IBD pathogenesis. A comprehensive understanding of ER stress’s role in intestinal injury and disease development will enhance the development of targeted therapeutic strategies for intestinal disorders (Fig. 3).

### IRE1-Xbp1 signaling pathway mediates inflammation

The IRE1-Xbp1 signaling pathway is a central mediator of inflammation, particularly within the intestinal environment. Its regulation is crucial for maintaining immune homeostasis and preventing excessive inflammatory responses associated with diseases like IBD. X-box binding protein 1(Xbp1), a key transcription factor in IRE1 signaling in response to ER stress and the UPR, is associated with IBD due to its location on chromosome 22q12 [73]. ER stress activates IRE1α, which binds to TRAF2 to form a complex that activates inhibitor of kappa B kinase. Activated inhibitor of kappa B kinase degrades IκB, releasing NF-κB [74, 75]. Additionally, IRE1α induces Thioredoxin interacting protein via microRNA intermediates. Thioredoxin interacting protein activates NLRP3 inflammatory vesicles, leading to Procaspase-1 cleavage and the maturation and secretion of IL-1β [76]. AP-1 is a crucial transcription factor that regulates the expression of various genes involved in inflammatory responses. It transcribes genes encoding tumor necrosis factor (TNF), keratinocyte growth factor, granulocyte–macrophage colony-stimulating factor, IL-8, and certain cytokine receptors [77]. The IRE1α-GSK3β axis plays a significant role in regulating inflammation through the mechanisms of ER stress. Glycogen synthase kinase 3 beta (GSK-3β) is recognized as a positive regulator of inflammatory processes, with its activity increasing under conditions of ER stress. While IRE1α-mediated GSK-3β activation regulates *IL-1β* gene expression, IRE1α-mediated Xbp1 splicing regulates *TNF-α* gene expression, highlighting the differential roles of these UPR mediators in ER stress-induced inflammation. The differential roles of GSK-3β and Xbp1 in mediating cytokine expression during ER stress highlight the complexity of inflammatory regulation [78]. Conditional deletion of Xbp1 in the intestinal epithelium leads to significant dysfunction in Paneth cells, characterized by the activation of IRE1α and JNK, which may involve downstream NF-κB. This cascade triggers an inflammatory response, as noted in various studies [6]. For instance, Lin et al. [79] observed signs of IBD in mice with intestinal deletion of Xbp1, including crypt loss, ulceration, and immune cell infiltration. These findings underscore the protective role of the IRE1-Xbp1 pathway in maintaining the intestinal mucosal barrier, preserving intestinal homeostasis, and inhibiting inflammatory processes.

### PERK-eIF2α-ATF4-CHOP signaling pathway mediates inflammation

The PERK-eIF2α-ATF4-CHOP signaling pathway is essential for regulating inflammation in response to ER stress. Its activation leads to both adaptive responses and potential apoptotic pathways, depending on the duration and severity of the stress. The activated PERK-eIF2α signaling pathway plays a significant role in mediating inflammation through translational arrest, which reduces the levels of IκBα, leading to the activation of NF-κB [80, 81]. As mentioned previously, ER stress induces the expression of the pro-inflammatory cytokine IL-8 and nucleoprotein translocation of CHOP. According to Park et al. [82] ER stress-induced CHOP inhibits the expression of peroxisome proliferator-activated receptor γ (PPARγ), a known negative regulator of NF-κB, thereby facilitating the activation of NF-κB. The modulatory effects of CHOP on PPARγ during ER stress significantly enhance NF-κB activation and IL-8 production in intestinal epithelial cells. Additionally, CHOP's role in promoting apoptosis through pathways involving Mac-1, ERO-1α, and caspase-11 further elucidates its contribution to inflammation and colitis development [83]. These findings highlight the dual role of CHOP in both promoting inflammation through NF-κB activation and potentially contributing to cellular apoptosis under prolonged ER stress conditions. Understanding this signaling pathway may provide insights into therapeutic strategies aimed at modulating inflammation associated with chronic diseases. Additionally, the activation of the IL-23/IL-17 axis is fundamentally linked to CD, as evidenced by elevated levels of IL-17 and IL-23 positive cells in affected tissues. This pathway plays a crucial role in mediating the inflammatory processes associated with CD [84]. Verstockt et al. [85] demonstrated that the interaction between the ER stress and Toll-like receptors (TLRs) agonists enhances the expression of the IL-23 p19 subunit mRNA and increases the secretion of IL-23 protein. The interaction between ER stress and TLR signaling pathways further enhances this inflammatory response, highlighting potential therapeutic targets for managing CD.

### The ATF6 signaling pathway mediates inflammation

The ATF6 signaling pathway plays a significant role in mediating inflammation, particularly in the context of ER stress and various inflammatory conditions. This pathway is part of the UPR, which is activated in response to ER stress and is crucial for maintaining cellular homeostasis. Like the other two branches of the UPR-IRE1α and PERK-ATF6 activates NF-κB signaling through transient phosphorylation of AKT, which enhances the expression of inflammatory cytokines and exacerbates intestinal inflammation. However, chronic ER stress results in the downregulation of AKT phosphorylation [86]. Shkoda et al. [87] observed that chronic inflammatory conditions in IBD are associated with elevated expression of GRP78 and activation of ER stress. Notably, they found that IL-10 inhibits the recruitment of ATF6 to the GRP78 promoter via the p38 MAPK pathway in IL-10-deficient mice. This finding suggests a direct mechanistic link between p38 MAPK and IL-10 in suppressing inflammation-induced ER stress. Furthermore, deletion or mutation of the S1P-encoding gene increases susceptibility to colitis, indicating a crucial role for the S1P-ATF6 axis in IBD pathogenesis [88–90]. Stengel et al. [91] demonstrated that impaired autophagy or unresolved ER stress in intestinal epithelial cells (IECs) led to increased activity of two upstream coactivators of ATF6α: Acyl-CoA synthetase long-chain family member 1 (ACSL1) and casein kinase 2 Beta (CSNK2B). This activation, in turn, triggered intestinal inflammation. Importantly, both in vitro and in vivo inhibition of ACSL1 or CSNK2B reduced pro-inflammatory signaling and cytokine secretion. These findings suggest that targeting upstream regulators of ATF6α could represent a novel therapeutic approach for inflammatory responses arising from impaired autophagy and ER stress in IECs.

### Autophagy mediates intestinal inflammation

Autophagy is a key regulator of intestinal inflammation through its roles in maintaining cellular homeostasis, regulating immune responses, interacting with gut microbiota, and controlling cytokine production. It has been indicated that the levels of LC3II, a marker of autophagy, were elevated in conditions associated with IBD, suggesting that autophagy is intricately involved in its pathogenesis [92]. Moreover, mutations in several autophagy-related genes, including *ULK1*, leucine-rich repeat kinase 2 gene (*LRRK2*), and nucleotide-binding and oligomerization domain 2 gene (*NOD2*), have been closely associated with the development of IBD [93–96]. These genetic alterations highlight the critical role that autophagy plays in maintaining intestinal health and regulating immune responses. Additionally, LRRK2 protein kinase was found to bind to and induce K48-linked ubiquitination of Beclin-1, a key autophagy promoter. This ubiquitination leads to the degradation of Beclin-1 and its phosphorylation, ultimately promoting intestinal inflammatory responses [97, 98]. Grizotte-Lake summarized the critical role of autophagy in IECs and its implications for the pathogenesis of IBD. Impaired autophagy in IECs has been shown to increase sensitivity to TNF-α-induced injury and cell death, which is significant in the context of IBD [99]. Furthermore, autophagy plays a critical role in the development, maintenance, and function of Paneth cells, specialized epithelial cells located at the base of intestinal crypts. These cells are essential for gut homeostasis, secreting antimicrobial peptides and maintaining the intestinal microbiota [100]. Research has emphasized the importance of autophagy-related proteins, particularly immunity-related GTPase M (IRGM) and its mouse homologue IRGM1, in regulating Paneth cell morphology and function [101]. Recent advancements have shed light on the regulation of autophagy in key cells involved in intestinal inflammation, such as IECs and immune cells. Autophagy modulators like vitamin D and rapamycin have emerged as potential therapeutic agents for intestinal inflammation [102]. Additionally, the discovery of novel autophagy modulators in the context of intestinal inflammation could pave the way for the development of innovative treatments for IBD (Fig. 3). Enhancing our understanding of how these modulators function can lead to targeted therapies that address inflammation and restore gut homeostasis through improved autophagic activity.

### The ER-stress-autophagy axis mediates intestinal inflammation

As previously discussed, ER stress can induce autophagy through multiple pathways, including the UPR and Akt signaling [103]. A complex interplay exists between unresolved ER stress, impaired autophagy, and pro-inflammatory signaling in IECs [104]. Increased NF-κB signaling and spontaneous intestinal inflammation in vivo have been observed in Xbp1- and Atg1611-deficient IECs with elevated IRE1α levels [105]. When ER stress intensifies in IECs, the inhibition of IKKα signaling can disrupt the balance of ATG16L1, a crucial autophagy-related protein. This imbalance contributes to the development of intestinal inflammation [106]. The ATG16L1 (T300A) variant and mutations in the *ATG16L1* gene are associated with significant alterations in Paneth cell function, particularly affecting their response to ER stress and regulation of IgA production by B cells [105, 107]. The simultaneous deletion of Atg16L1 and the ER stress transcription factor *Xbp1* led to a dramatic reduction in Paneth cell numbers and exacerbated intestinal inflammation [108]. In IECs, ATF6α serves as a crucial intermediary signaling molecule that regulates both the upstream factor (Xbp1 and the downstream autophagy-related molecule Atg16L1. ER stress activates autophagy and facilitates the translocation of mAtg9 through ATF6-mediated upregulation of Death-associated protein kinase 1 [109]. Impaired autophagy can lead to an increase in ER stress. Inhibiting trigger receptor-1 expression on myeloid cells has been shown to increase the levels of key autophagy proteins like Atg5, Atg16L1, and CMA. This restoration of autophagy, in turn, reduces the levels of ER stress markers such as PERK, IRE-1α, and ATF-6α. Additionally, it helps to mitigate intestinal dysbiosis and alleviate ER stress. These insights point to TREM-1 as a novel therapeutic target for managing IBD by enhancing autophagic processes and alleviating ER stress [109]. The intricate interplay between ER stress and autophagy plays a significant role in regulating intestinal inflammation. While ER stress can trigger autophagy, leading to a reduction in pro-inflammatory cytokines and improved intestinal barrier function, thereby mitigating intestinal damage. Conversely, impaired autophagy can exacerbate apoptosis and further intensify ER stress, contributing to the development of IBD. Understanding these mechanisms offers potential therapeutic targets for managing inflammatory conditions in the gut by enhancing autophagic processes or alleviating ER stress. These insights underscore the importance of maintaining a balance between ER stress and autophagy to promote intestinal health and prevent inflammatory diseases like IBD (Fig. 3).

## The close relationship between gut microbes and intestinal inflammation

Gut microbes play a pivotal role in modulating intestinal inflammation through their interactions with the immune system, production of metabolites, and maintenance of barrier function. Dysbiosis not only contributes to inflammatory diseases like IBD but also highlights potential therapeutic targets for managing these conditions effectively. The animal gut microbiota, comprising over 100 trillion microorganisms, plays a crucial role in maintaining the health and functionality of the host’s intestinal system. Under normal conditions, these microorganisms establish a dynamic ecological balance with the host and the external environment. However, disturbances to this balance known as dysbiosis can lead to a cascade of adverse effects, including impaired barrier function, inflammation, and compromised immune response, ultimately contributing to the development of various diseases [110]. Yet, numerous studies have demonstrated that ameliorating colitis, particularly UC, involves a multifaceted approach, encompassing the repair of the intestinal mucosal barrier, reduction of systemic inflammation, and restoration of the gut microbiota [111–113].

**Fig. 4:**
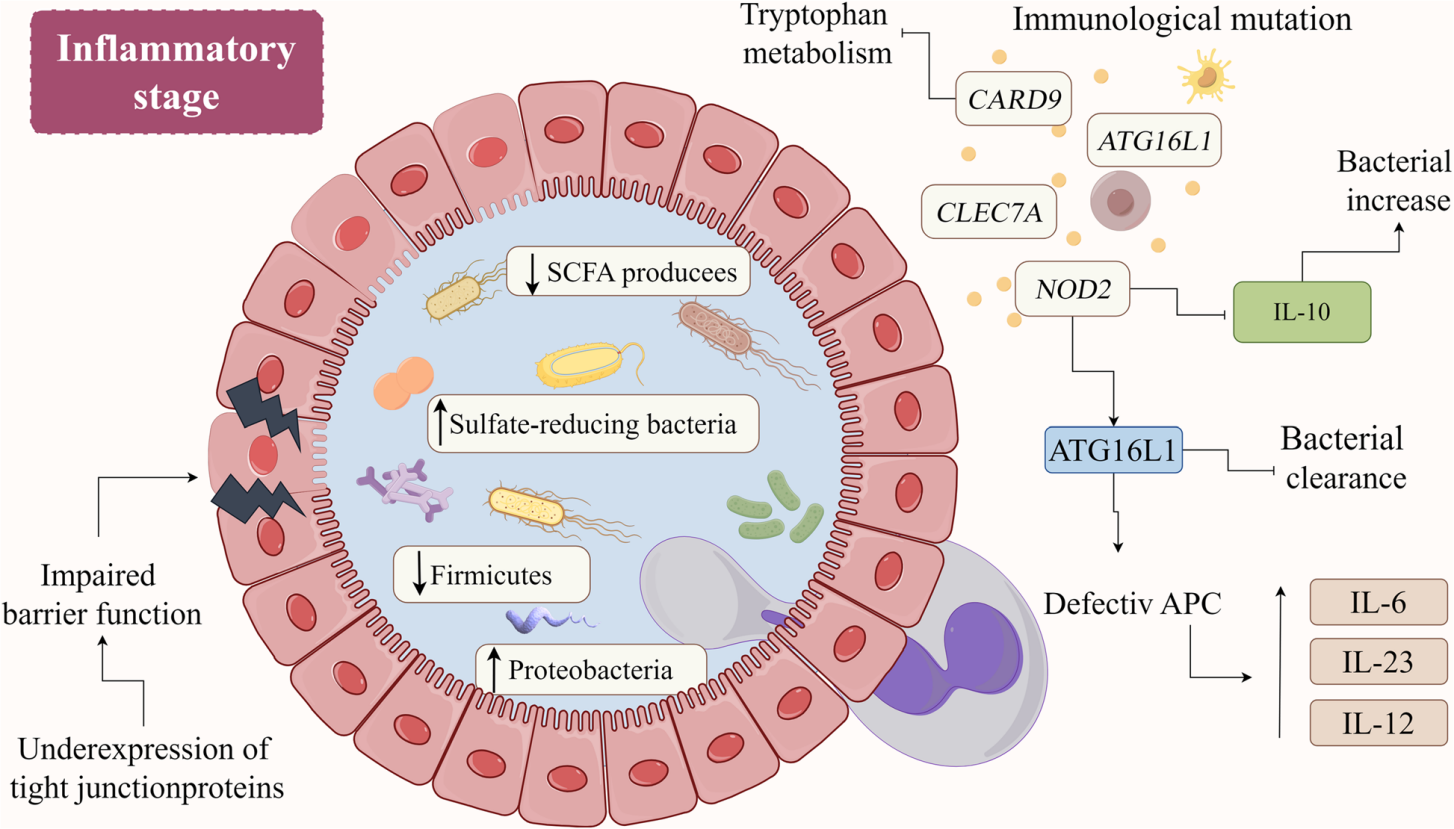
Gut microbiota and inflammatory bowel disease. Gut microbiota dysbiosis, characterized by alterations in microbial composition and function, is strongly associated with IBD. A decrease in beneficial bacteria like Firmicutes and an increase in harmful bacteria like Proteobacteria contribute to disrupted intestinal barrier function and inflammation. Additionally, reduced short-chain fatty acid production and increased sulfate-reducing bacteria further exacerbate the inflammatory response. Genetic factors also play a significant role in IBD pathogenesis. Mutations in genes such as *NOD2*, *ATG16L1*, *CARD9*, and *CLEC7A* impair the immune response and enhance susceptibility to IBD. For instance, *NOD2* mutations are linked to decreased IL-10 production and increased mucosal bacterial colonization. Moreover, the interaction between *NOD2* and *ATG16L1* is crucial for bacterial clearance and antigen presentation. Disruptions in this interaction, caused by mutations in either gene, can lead to increased pro-inflammatory cytokine secretion and exacerbated gut inflammation. *ATG16L1,* autophagy-related 16-like 1; *CARD9,* caspase recruitment domain-containing protein 9; *CLEC7A*, C-type lectin domain family 7 member A; IBD, inflammatory bowel disease; IL, interleukin; *NOD2,* nucleotide-binding and oligomerization domain 2 gene; SCFAs, short-chain fatty acids

The global incidence of IBD is steadily increasing, with UC and CD as the most prevalent forms. Emerging evidence strongly suggests a critical role of intestinal dysbiosis, characterized by reduced microbial diversity and altered microbial composition, in the pathogenesis of IBD [114, 115]. Short-chain fatty acids (SCFAs) are reduced in individuals with IBD. These fatty acids serve as an energy source for colonic epithelial cells and promote the expansion of regulatory T cells within the colon, contributing to gut health and immune function [116]. Furthermore, individuals with IBD exhibit an elevated abundance of sulfate-reducing bacteria. The hydrogen sulfide produced by these bacteria has detrimental effects on colonic health, particularly by inhibiting the utilization of butyrate, a critical energy source for colonic epithelial cells [117]. Furthermore, ecological dysregulation of gut microbiota can lead to immune dysregulation and promote inflammatory responses in IBD. Genetic factors, such as mutations in genes involved in immune function, including nucleotide-binding oligomerization domain 2 (*NOD2*), autophagy-related 16-like 1 (*ATG16L1*), caspase recruitment domain-containing protein 9 (*CARD9*), and C-type lectin domain family 7 member A (*CLEC7A*), can exacerbate these inflammatory responses in IBD [118]. NOD2 and ATG16L1 proteins can interact with each other. Genetic mutations affecting either protein can impair this interaction, hindering bacterial clearance and antigen presentation. This impaired function can increase pro-inflammatory cytokine secretion and lead to chronic inflammation [119–121]. Additionally, a study has demonstrated that impaired microbial tryptophan metabolism can contribute to colitis susceptibility due to the reduced activation of aryl hydrocarbon receptor, a critical regulator of intestinal immunity that is activated by various tryptophan metabolites [122] (Fig. 4).

### The gut microbe-ER stress axis regulates gut inflammation

**Fig. 5:**
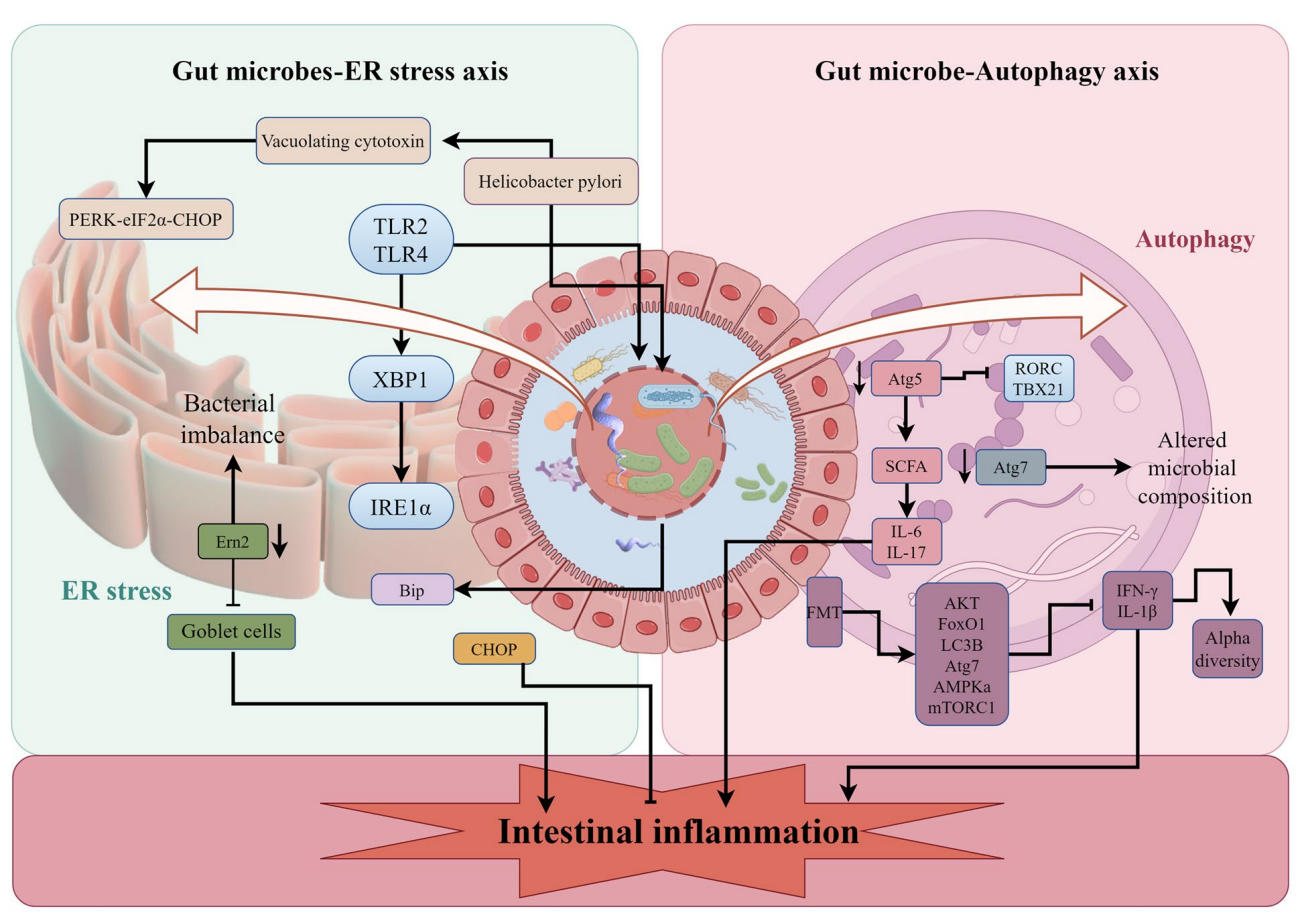
Gut microbe-ER stress and autophagy axes in intestinal inflammation. Gut microbe-ER stress axis. Gut microbes can directly influence ER stress, leading to intestinal inflammation. For example, *Ern2* gene-deficient mice exhibit dysregulated gut microbiota, impaired goblet cell development, and increased susceptibility to colitis. TLRs act as crucial links between the gut microbiota and ER stress. TLRs recognize microbial components and activate signaling pathways, including the IRE1α-XBP1 pathway, which can induce ER stress. Certain gut microorganisms, such as *Helicobacter pylori*, can trigger ER stress through virulence factors like VacA, leading to activation of the PERK-eIF2α-CHOP pathway. Conversely, other microorganisms may inhibit ER stress by modulating the expression of ER chaperone proteins like Bip or the pro-apoptotic factor CHOP. Gut microbe-autophagy axis. The gut microbiota also interacts with autophagy, another cellular process involved in maintaining cellular homeostasis and mitigating inflammation. Disruptions in autophagy, such as genetic deletions of Atg7 or Atg5, can lead to alterations in gut microbiota composition, decreased short-chain fatty acid production, increased pro-inflammatory cytokine levels, and upregulation of IBD-associated transcription factors like RORC and TBX21. FMT can influence autophagy in the intestinal mucosa. While FMT can increase the levels of autophagy-associated proteins, it can also exacerbate intestinal inflammation and alter the gut microbiota composition, potentially by increasing microbial diversity and altering the structure of the colonic flora. AMPK, adenosine monophosphate-activated protein kinase; CHOP, C/EBP Homologous Protein; eIF2α, eukaryotic initiation factor 2 alpha; ER, endoplasmic reticulum; FMT, fecal microbiota transplantation; IBD, inflammatory bowel disease; IL, interleukin; IRE1α, inositol-requiring enzyme 1α; mTORC1, mammalian target protein 1 of rapamycin; PERK, protein kinase R-like ER kinase; SCFAs, short-chain fatty acids; TLRs, toll-like receptors; XBP1, X-box binding protein 1

The gut microbe-ER stress axis is a critical regulatory pathway influencing gut inflammation and IBD pathogenesis [123]. Impaired microbial metabolism can exacerbate ER stress responses in IECs, leading to increased production of pro-inflammatory mediators and disruption of tight junctions, compromising gut barrier function [124]. This ultimately contributes to chronic inflammation and increased susceptibility to bacterial invasion [125, 126]. ERN2/IRE1β, a paralog of the ER stress kinase sensor ERN1/IRE1α, is uniquely expressed in epithelial cells lining the mucosal surfaces of the gastrointestinal and respiratory tracts [105]. Grey et al. [127] demonstrated that ERN2-deficient mice exhibited gut microbiota dysregulation, impaired goblet cell development, and increased susceptibility to colitis. These findings suggest that the ER transmembrane protein ERN2 mediates gut microbial-colonic crosstalk and contributes to host defense mechanisms. TLRs, such as TLR4 and TLR2, can connect the ER stress response to microbial stimuli. These TLRs can activate the ER stress sensor IRE1α and its downstream target Xbp1 [128]. TLRs, such as TLR4 and TLR2, can connect the ER stress response to microbial stimuli [129]. These TLRs can activate the ER stress sensor IRE1α and its downstream target Xbp1. Gastrointestinal microorganisms can induce ER stress and the UPR. For instance, *Helicobacter pylori* can trigger ER stress in gastric epithelial cells through its vacuolating cytotoxin (VacA). VacA activates the PERK-eIF2α-CHOP pathway, leading to mitochondrial damage and subsequent apoptosis [130]. *Listeria monocytogenes* can activate all three branches of the UPR and induce apoptotic signaling through its cytolysin listeriolysin O, which disrupts ER homeostasis without cellular entry [131]. Cholera toxin upregulates the ER chaperone Bip [132]. Pore-forming toxins produced by various bacteria, including *Staphylococcus aureus*, *Streptococcus pyogenes*, *Clostridium perfringens*, and *Aeromonas hydrophila*, activate the IRE1-Xbp1 pathway in response to PFT-induced ER stress, serving as a defense mechanism [133]. Beyond pathogenic bacteria, commensal bacteria can also modulate ER stress. *Bacillus subtilis* HW2 can inhibit intestinal ER stress, enhance microbial diversity, and improve barrier function, reducing inflammation in broilers [126]. *Lactobacillus johnsonii* alleviates ER stress by reducing CHOP expression and inhibiting ER stress-induced apoptosis, thereby mitigating colitis [134]. Additionally, gut microbial metabolites, such as N-acylated dipeptide aldehydes and bisindole methanes, can regulate ER stress by targeting specific pathways like by inhibiting proteases or interfering with lipid metabolism [135]. In addition, *Lactobacillus acidophilus* (LA1) can alleviate ER stress, inhibit NF-κB activation, and reduce intestinal inflammation [136]. In conclusion, the gut microbe-ER stress axis plays a crucial role in intestinal health and disease. By influencing ER stress responses, gut microbes can modulate gut inflammation, immune function, and barrier integrity (Fig. 5).

### Gut microbe-autophagy axis regulates intestinal 

The gut microbe-autophagy axis is a crucial regulatory pathway that influences intestinal inflammation and homeostasis. The intricate interplay between the gut microbiota and autophagic processes highlights the importance of a balanced microbial environment in supporting effective immune responses and preventing chronic inflammation. Autophagy plays a crucial role in regulating the composition of the gut microbiota, and its impairment is linked to gut dysbiosis. Research indicates that basal autophagy levels are significantly reduced in the colonic epithelium of germ-free mice compared to their conventionally raised counterparts [137]. The *Atg16L1* gene, a marker for autophagy, has been associated with CD, suggesting that autophagy is instrumental in mediating host-microbe interactions within the gut [138, 139]. Impaired autophagy alters the colonization patterns of gut microbiota, disrupting their role as a physical barrier essential for maintaining gut immune homeostasis. Animal studies have demonstrated that deletion of *Atg16L1* in macrophages leads to a pro-inflammatory metabolic state characterized by impaired mitochondrial function and NLRP3 inflammasome activity [140, 141]. Furthermore, specific deletion of *Atg7* in the mouse colonic epithelium results in significant changes to fecal microbiota composition, including an increase in total bacterial counts and an enrichment of species such as *Clostridium leptum*, *Eubacterium cylindroides*, and *Bacteroides fragilis* [142]. Additionally, reductions in *Lachnospiraceae* and *Ruminococcaceae* abundance have been observed in *Atg5*-deficient mice, correlating with decreased production of SCFAs. Conversely, an increase in abundance in the jejunum-ileum has been linked to the recruitment and activation of inflammatory cells, leading to elevated production of pro-inflammatory cytokines [143]. The gut microbiome can induce adaptive immune responses that can lead to chronic inflammation [144]. While autophagy is known to regulate inflammatory processes, it has been hypothesized that autophagy's regulation of inflammation may influence the composition of the gut microbiota, a bidirectional relationship between the gut microbiota and autophagy, referred to as the microbial-autophagy axis [145]. Fecal microbiota transplantation (FMT) has emerged as a promising therapeutic approach that can modulate this axis. In piglet models, FMT has been shown to increase levels of autophagy-associated proteins in the intestinal FoxO pathway. This includes proteins such as AKT, Forkhead Box Protein O1, Forkhead Box O3, LC3B, Atg7, AMPKα, and mTORC1 within the AMPK-mTOR pathway. Additionally, FMT decreased levels of pro-inflammatory cytokines such as gamma interferon and IL-1β. Notably, FMT also increased the α-diversity of intestinal microbes and modified the structure of the colonic flora, indicating that FMT triggers autophagy in the intestinal mucosa and alleviates intestinal damage caused by *Escherichia coli* K88 [146]. The intricate interplay between gut microbes and autophagy, termed the microbial-autophagy axis, plays a pivotal role in regulating gut inflammation. This bidirectional relationship highlights the significant impact of gut microbial composition on autophagy regulation, which in turn influences inflammatory responses and overall gut health. Continued research into this axis could provide valuable insights into developing novel therapeutic strategies targeting both the gut microbiota and autophagy to alleviate chronic inflammatory conditions (Fig. 5).

## Therapeutic strategies based on ER stress, autophagy and gut microbes mediated intestinal inflammation

Therapeutic strategies targeting the interplay between ER stress, autophagy, and gut microbiota present a novel approach to managing intestinal inflammation associated with IBD.

### Plant extracts

Plant extracts are increasingly recognized for their potential to combat drug resistance and alleviate side effects associated with conventional medications. These extracts have also shown promise in addressing ER stress-induced intestinal inflammation, which is a significant concern in various gastrointestinal disorders. Pterostilbene, a naturally occurring compound, has demonstrated significant efficacy in alleviating ER stress, restoring autophagic flux and gut microbiota [147]. This effect is mediated through the downregulation of GRP78 and CHOP in the colon of IUGR piglets [147]. Additionally, pterostilbene upregulates the expression of Beclin 1 and enhances the LC3 II/I ratio, indicating an increase in autophagic activity. Moreover, pterostilbene has been shown to ameliorate the insufficient secretion of mucin 2 and trefoil factor 3 in the colons of IUGR piglets, thereby improving intestinal barrier function [147]. These findings suggest that pterostilbene may serve as a therapeutic agent for conditions associated with ER stress and impaired autophagy in intestinal inflammation [147]. Quercetin, a flavonoid abundant in various fruits and vegetables, has been demonstrated to mitigate damage to both the pancreas and ileum in acute necrotizing pancreatitis. This protective effect is associated with improved intestinal barrier function and reduced inflammatory responses. Quercetin achieves this by inhibiting the TLR4-MyD88-p38 MAPK signaling pathway and alleviating ER stress [148]. Berberine (BBR), a potent anti-inflammatory and immunomodulatory agent derived from *Coptis chinensis*, is a crucial component in the treatment of IUGR animals. Yan et al. [149] reported that BBR reduced colonic *GRP78* mRNA levels, inhibited the caspase-12/caspase-3 apoptotic signaling pathway, alleviated clinical symptoms like diarrhea, and mitigated colonic histopathological damage, including inflammatory cell infiltration, epithelial edema, and disrupted epithelial structures in the colonic mucosa. *Magnolia officinalis* cortex (MO, Hou po in Chinese) has been shown to reduce the expression of inflammatory cytokines in zebrafish intestines. This effect is mediated through inhibition of the PI3K/AKT/NF-κB signaling pathway. Additionally, MO has been observed to increase the abundance of beneficial gut bacteria such as *Lactobacillus*, *Blautia*, and *Saccharomyces*, while decreasing the levels of harmful bacteria like *Plesiomonas* and *Aeromonas*. These changes in gut microbiota composition contribute to alleviated intestinal inflammation and overall gut health [150]. Supplementation with appropriate amounts of curcumin in animal models of IBD has been shown to increase the relative abundance of *Lactobacillus* species. This increase in beneficial bacteria contributes to improved mucosal immunity by enhancing the production of secretory immunoglobulin A, an immunoglobulin crucial for maintaining intestinal microbial balance and barrier function [151].

### Amino acid

Amino acids are essential nutrients that play crucial roles in both host cell functions and the metabolism of gut bacteria. Their significance extends beyond mere nutrition, as they also exhibit anti-inflammatory properties, particularly in the context of gut health [152]. L-glutamine and L-arginine are essential amino acids that regulate IECs proliferation and differentiation [153, 154]. Supplementation with L-glutamine and glycine has been shown to increase tight junction protein expression [155], improve intestinal barrier function, and alleviate ER stress and apoptosis in IECs [156]. Additionally, L-glutamine has been reported to regulate tight junction protein permeability and enhance intestinal mucosal barrier function by activating the CaMKK2-AMPK signaling pathway [157]. Compared to other amino acids, glycine (Gly) exhibits potent antioxidant and anti-inflammatory properties. Gly promotes glutathione synthesis, which helps mitigate oxidative stress. Additionally, Gly can regulate intestinal tight junction proteins, thereby inhibiting inflammatory responses and ER stress [158]. Zhang et al. [159] demonstrated that Gly alleviated *Citrobacter* *rodentium*-induced colitis by inhibiting the ATF6α-CHOP signaling pathway. Glutathione, a key antioxidant, plays a crucial role in regulating ER protein folding [160]. Reduced levels of γ-glutamylcysteine and glutathione have been associated with ER stress [161]. Engevik et al. [162] found that *Bifidobacterium dentium*-derived γ-glutamylcysteine inhibited TNBS-induced colitis, reduced ER molecular chaperone expression, and inhibited ER stress-driven reactive oxygen species production. Additionally, amino acids can modulate the gut microbiota. Zhang et al. [163] showed that dietary supplementation with L-arginine or *N*-carbamylglutamate in IUGR lambs increased the abundance of Firmicutes, decreased the abundance of *Escherichia* and *Proteobacteria*, and alleviated oxidative stress and inflammation in the colon.

### Others

*Lactobacillus johnsonii* (LS31) is a probiotic strain that significantly alleviates intestinal inflammation through multi-target regulatory mechanisms [164]. LS31 inhibits the activation of the NOD1/2-RIP2 pathway and mitigates ER stress, thereby reducing NF-κB-dependent inflammatory responses and cellular damage. Furthermore, LS31 promotes the degradation of autophagosomes, facilitating the clearance of intracellular *Salmonella* and restoring autophagic flux, which limits pathogen proliferation [164]. These findings underscore the potential of LS31 as a multifaceted therapeutic agent for managing intestinal inflammation. Simultaneously, pharmacological intervention constitutes a critical aspect in the management of intestinal inflammation [103]. Certain therapeutic agents exert their effects by modulating ER stress. For instance, low-dose naltrexone has been shown to decrease ER levels and restore intestinal mucosal barrier integrity [103]. Furthermore, some pharmacological agents exhibit dual regulatory effects on both ER stress and autophagy. For example, extracellular vesicles derived from Lactobacillus alleviate inflammation by activating ER stress, thereby maintaining intestinal homeostasis [165]. Azathioprine, utilized in the treatment of IBD, induces autophagy through modulation of mTORC1 signaling transduction and the UPR sensor PERK [166].

As a limitation of this review and the current research, although some potential treatment strategies are mentioned, there is a lack of in-depth exploration of the mechanisms of targeted intervention in ER stress, autophagy, and gut microbiota, such as how to design more effective drugs or dietary supplements. Therefore, future studies on targeted interventions that address these interconnected pathways will be further considered in future research.

## Conclusion

The ER can alleviate stress and restore homeostasis through UPR. However, prolonged ER stress can lead to cellular damage, including autophagy and apoptosis. Chronic inflammation, a hallmark of IBD, is often associated with dysregulated ER stress and UPR signaling pathways, which can exacerbate inflammatory responses. Impaired autophagy exacerbates intestinal inflammation. The ER stress-autophagy axis plays a crucial role in regulating intestinal inflammation. The gut microbiota is a significant driver of intestinal inflammation, and both the gut microbe-ER stress axis and the gut microbe-autophagy axis contribute to the pathogenesis of intestinal inflammation in animals. By investigating the individual and synergistic regulation of gut inflammation by ER stress, autophagy, and gut microbiota, we can gain a deeper understanding of the pathogenesis of gut inflammation. This knowledge can enhance the development of novel therapeutic strategies targeting these pathways, potentially leading to more effective treatments for conditions like UC and CD. Future studies focusing on targeted interventions that address these interconnected pathways are essential for identifying specific intervention targets in gut-related inflammatory disorders.

## Data Availability

Not applicable.

## Abbreviations

ACSL1: Acyl-CoA synthetase long-chain family member 1
AMPK: Adenosine monophosphate-activated protein kinase
ATF: Activating transcription factor
ATG16L1: Autophagy-related 16-like 1
AP-1: Activator protein-1
BBR: Berberine
CARD9: Caspase recruitment domain-containing protein 9
CD: Crohn’s disease
CHOP: C/EBP homologous protein
CLEC7A: C-type lectin domain family 7 member A
CMA: Chaperone-mediated autophagy
CSNK2B: Casein kinase 2 beta
DAPK: Death-associated protein kinase
eIF2α: Eukaryotic initiation factor 2 alpha
ER: Endoplasmic reticulum
ERO1α: ER oxidoreductin 1 alpha
FMT: Fecal microbiota transplantation
GPX4: Glutathione peroxidase 4
GSK-3β: Glycogen synthase kinase 3 beta
HSP5A: Heat shock protein family A
IL: Interleukin
IBD: Inflammatory bowel disease
IECs: Intestinal epithelial cells
IRGM: Immunity-related GTPase M
MO: Magnolia officinalis cortex
mTORC1: Mammalian target protein 1 of rapamycin
NOD2: Nucleotide-binding oligomerization domain 2
PPARγ: Peroxisome proliferator-activated receptor γ
S1P: Site-1 protease
SCFAs: Short-chain fatty acids
TLR: Toll-like receptor
TNF: Tumor necrosis factor
TRAF2: Receptor-associated factor 2
UC: Ulcerative colitis
UPR: Unfolded protein response
Xbp1: X-box binding protein 1
